# *Leishmania* Mitochondrial Peroxiredoxin Plays a Crucial Peroxidase-Unrelated Role during Infection: Insight into Its Novel Chaperone Activity

**DOI:** 10.1371/journal.ppat.1002325

**Published:** 2011-10-27

**Authors:** Helena Castro, Filipa Teixeira, Susana Romao, Mariana Santos, Tânia Cruz, Manuela Flórido, Rui Appelberg, Pedro Oliveira, Frederico Ferreira-da-Silva, Ana M. Tomás

**Affiliations:** 1 IBMC - Instituto de Biologia Molecular e Celular, Universidade do Porto, Porto, Portugal; 2 ICBAS - Instituto de Ciências Biomédicas Abel Salazar, Universidade do Porto, Porto, Portugal; Washington University School of Medicine, United States of America

## Abstract

Two-cysteine peroxiredoxins are ubiquitous peroxidases that play various functions in cells. In *Leishmania* and related trypanosomatids, which lack catalase and selenium-glutathione peroxidases, the discovery of this family of enzymes provided the molecular basis for peroxide removal in these organisms. In this report the functional relevance of one of such enzymes, the mitochondrial 2-Cys peroxiredoxin (mTXNPx), was investigated along the *Leishmania infantum* life cycle. mTXNPx null mutants (*mtxnpx^−^*) produced by a gene replacement strategy, while indistinguishable from wild type promastigotes, were found unable to thrive in a murine model of infection. Unexpectedly, however, the avirulent phenotype of *mtxnpx^−^* was not due to lack of the peroxidase activity of mTXNPx as these behaved like controls when exposed to oxidants added exogenously or generated by macrophages during phagocytosis *ex vivo*. In line with this, *mtxnpx^−^* were also avirulent when inoculated into murine hosts unable to mount an effective oxidative phagocyte response (B6.p47^phox−/−^ and B6.RAG2^−/−^ IFN-γ^−/−^ mice). Definitive conclusion that the peroxidase activity of mTXNPx is not required for parasite survival in mice was obtained by showing that a peroxidase-inactive version of this protein was competent in rescuing the non-infective phenotype of *mtxnpx^−^*. A novel function is thus proposed for mTXNPx, that of a molecular chaperone, which may explain the impaired infectivity of the null mutants. This premise is based on the observation that the enzyme is able to suppress the thermal aggregation of citrate synthase *in vitro*. Also, *mtxnpx^−^* were more sensitive than controls to a temperature shift from 25°C to 37°C, a phenotype reminiscent of organisms lacking specific chaperone genes. Collectively, the findings reported here change the paradigm which regards all trypanosomatid 2-Cys peroxiredoxins as peroxide-eliminating devices. Moreover, they demonstrate, for the first time, that these 2-Cys peroxiredoxins can be determinant for pathogenicity independently of their peroxidase activity.

## Author Summary

*Leishmania* are insect-borne protozoan parasites that cause leishmaniasis, a spectrum of diseases with relevance in men and dogs and for which there are no adequate means of control. In order to successfully infect their mammalian hosts, *Leishmania* requires the expression of a set of key proteins. Here, we identify one mitochondrial protein of the parasite as one of such molecules. Interestingly, however, we also discovered that the reason why this enzyme is essential is not due to its well characterized peroxidase activity, which defines it as a member of the peroxiredoxin family. Instead, the importance of this protein during infection may derive from its ability to function as a chaperone, as implied here. In short, by proposing a novel, peroxidase-unrelated role for the *Leishmania* mitochondrial peroxiredoxin during mammals' infection, this work opens new perspectives regarding the physiologic function of this enzyme.

## Introduction

The last 15 years have contributed decisively towards the dissection of the enzymatic pathways that lead to peroxide elimination in trypanosomatids, a group of organisms that includes *Leishmania* spp., *Trypanosoma brucei* and *Trypanosoma cruzi*, the causative agents of the different manifestations of leishmaniasis, African sleeping sickness and Chagas' disease, respectively. In these protozoan parasites, which lack the highly efficient enzymes catalase and selenium-containing glutathione peroxidases (GPXs), members of the peroxiredoxin family are regarded as key elements of the peroxide-reduction machinery [1].

Peroxiredoxins (PRXs) are ubiquitous enzymes that use a redox active cysteine residue (peroxidatic Cys) to reduce a broad spectrum of substrates, namely H_2_O_2_, organic hydroperoxides and peroxynitrite (ONOO^−^). Upon reduction of the peroxide, the peroxidatic Cys-SH is oxidized to sulfenic acid (Cys-SOH). In PRXs harboring two active cysteines (known as 2-Cys PRXs), the sulfenic acid is reduced by another Cys residue (resolving Cys) to form a disulfide. According to the location of the resolving Cys, 2-Cys PRXs can be classified as typical or atypical [2]. In trypanosomatids all PRXs characterized to date fall in the category of typical 2-Cys [3]. Peroxiredoxins return to their reduced state upon reduction of the disulfide by an appropriate electron donor. In trypanosomatids such reductant is a unique oxidoreductase of the thioredoxin superfamily, known as tryparedoxin [4], which itself is reduced by these organisms' specific thiol trypanothione [*N* ^1^,*N* ^8^-bis(glutathionyl)spermidine; 1,3]. For that reason trypanosomatid PRXs are commonly referred to as tryparedoxin peroxidases or TXNPxs. Apart from participating in antioxidant defense, members of the 2-Cys PRX subfamily are increasingly recognized as playing a more subtle and sophisticated role as regulators of peroxide-mediated cell signaling [2]. More recently, a function as molecular chaperones has also been proposed for some of these enzymes [5], [6].

In trypanosomatids 2-Cys PRXs are present in the parasites' cytosol and single mitochondrion [1]. Cytosolic 2-Cys PRXs (or cTXNPxs) are believed to work as general antioxidant devices that minimize the oxidative insult generated by the parasites' host. In agreement with this, overexpression of cTXNPx in *L. infantum* and *T. cruzi* was found to confer resistance to H_2_O_2_ and peroxynitrite of exogenous origin [7]–[12], while down-regulation of the *T. brucei* counterpart enhanced sensitivity to bolus H_2_O_2_ [13]. This peroxidase activity should be particularly relevant for *Leishmania* and *T. cruzi*, which can invade and proliferate in phagocytes. Not surprisingly, cTXNPxs of these organisms were pointed out as important virulence factors [12], [14]–[17].

Mitochondrial TXNPxs (or mTXNPxs) are, due to their location, favorably positioned to eliminate peroxides produced endogenously, namely those formed as by-products of oxidative phosphorylation, and this was argued to be their main function [7], [8], [11]. However, the observation that overexpression of mTXNPx confers protection towards exogenously added and macrophage-derived H_2_O_2_ and peroxynitrite [7], [8], [10], [11], suggested that these enzymes might also contribute to shield trypanosomatids from the oxidative challenge induced by their hosts. This assumption was strengthened by the observation that increased mTXNPx expression is associated with virulent *T. cruzi* phenotypes [17]. Apart from functioning as general antioxidant devices, the *Leishmania donovani* mTXNPx was reported to prevent H_2_O_2_-induced programmed cell death [18]. One additional, peroxidase-related role suggested for mTXNPxs was regulation of kinetoplast DNA (kDNA) replication. The kDNA is a network of catenated maxi and mini DNA circles that compose the mitochondrial DNA of trypanosomatids and whose replication is initiated when the universal minicircle sequence binding protein (UMSBP) binds specific sequences on minicircle DNA [19]. UMSBP binds to the DNA when it is reduced and is released when oxidized. According to the proposed model, mTXNPx would oxidize UMSBP [20].

The present study aimed at dissecting the functional relevance of mTXNPx in *Leishmania infantum*. *Leishmania* have a digenic life cycle that includes two morphologically and physiologically distinct stages: the promastigote (an extracellular form residing in the insect vector) and the amastigote (an intracellular form living inside the mammalian host). Using a homozygous knockout *L. infantum* line unable to express mTXNPx it was found that, while redundant in promastigotes, this mitochondrial 2-Cys PRX is essential for the establishment of a successful infection in mammals. This result establishes mTXNPx as factor determinant for *Leishmania* pathogenicity. Importantly, the data gathered here indicate that the essential role played by mTXNPx is not related to its peroxidase activity. Rather, the decreased infectivity of the mutants may be explained by the ability of mTXNPx to function as a chaperone, an activity disclosed here.

## Results

### Generation of *mTXNPx* mutants

**Figure 1:**
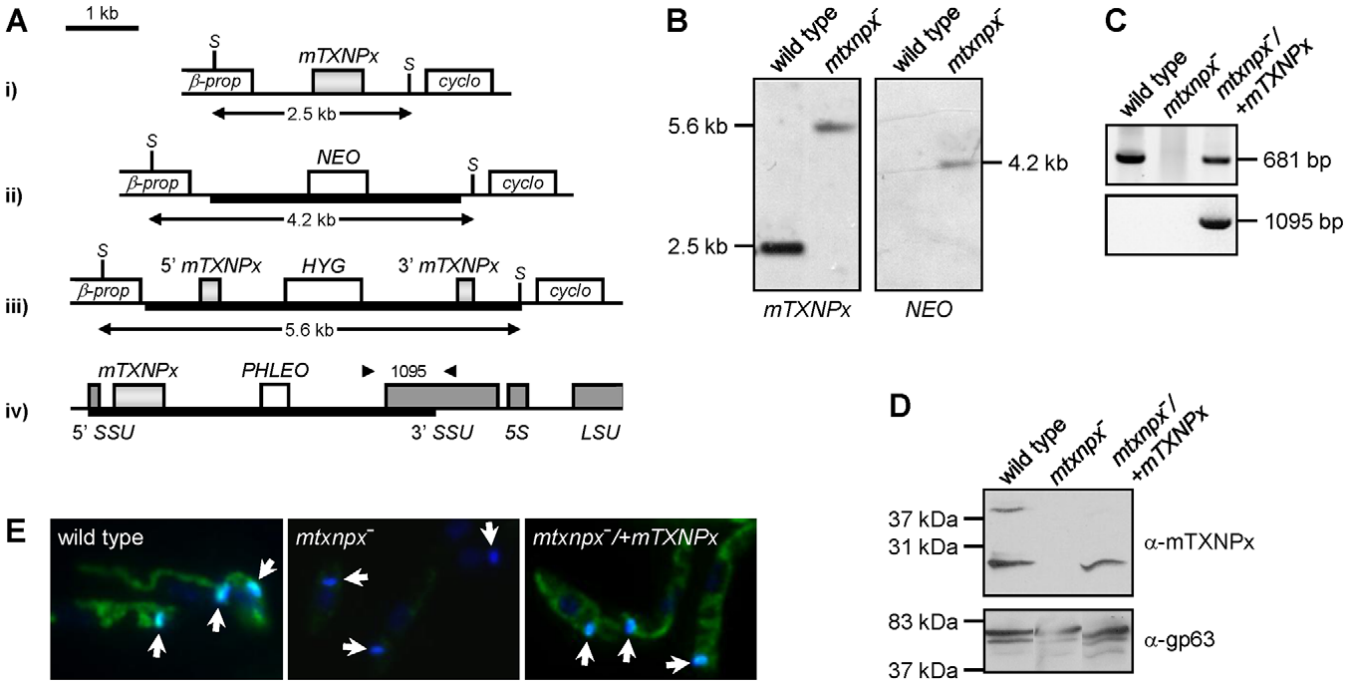
Generation of *mTXNPx* mutants. A. Genomic organization of the *mTXNPx locus* with its flanking β-propeller (*β-prop*) and cyclophilin (*cyclo*) genes in i) wild type and in ii) *NEO*- and iii) *HYG*- targeted alleles. The *HYG* disruption construct eliminated 144 nt of the *mTXNPx* coding sequence, leaving intact the first 292 and the last 245 nt (light grey boxes). *Sac*I (S) restriction sites are indicated. Scheme iv) represents the small subunit 18S rRNA (SSU) *locus* of *mtxnpx^−^*/+*mTXNPx* mutants with the integrated *PHLEO* construct harboring the *mTXNPx* coding sequence. Dark grey boxes represent the 5′ and 3′ coding regions of SSU, of 5S rRNA and of the large subunit 28S rRNA (LSU). Thick lines represent the integrated DNA constructs. Arrowheads show the location of primers used in PCR analysis (in C.) to diagnose for the correct integration of the *PHLEO* cassette into the ribosomal *locus*. The number in between arrowheads refers to the expected size of the corresponding PCR product. B. Southern blot analysis of *Sac*I-digested genomic DNA of wild type and *mtxnpx^−^* mutants, hybridized with *mTXNPx* and *NEO* ORFs. In *mtxnpx^−^*, the 5.6 kb band revealed by the *mTXNPx* probe corresponds to the gene disrupted by the *HYG* cassette. C. PCR analysis of genomic DNA from wild type, *mtxnpx^−^* and *mtxnpx^−^/+mTXNPx* parasites, using specific primers to amplify *mTXNPx* ORF (681 bp) or to diagnose for the correct integration of the *PHLEO* cassette into the ribosomal *locus* (1095 bp; primer location represented in A.). D. Western blot analysis of wild type, *mtxnpx^−^* and *mtxnpx^−^/+mTXNPx* promastigotes, incubated with the anti-mTXNPx antibody and, upon stripping, with anti-gp63 antibody (control for loading). The band above 37 kDa in the upper panel is dimeric mTXNPx. E. Indirect immunofluorescence of parasites lines as above, incubated with anti-mTXNPx antibody (green), merged with DAPI (blue). 1000× magnification. The kDNA is indicated by arrows.

To study the relevance of mTXNPx during the *L. infantum* life cycle, a mutant parasite line unable to express this enzyme was produced by homologous recombination. Since *Leishmania* has a diploid genome, two successive rounds of gene targeting were required to obtain homozygous knockout mutants. The first *mTXNPx* allele was replaced by a *NEO* integration cassette, while the second was disrupted with a *HYG* construct (Figure 1A). As confirmed by Southern blot (SB) of the double transfectants, both *mTXNPx* alleles were successfully targeted, thus resulting in the generation of a Δ*mtxnpx::NEO*/Δ*mtxnpx::HYG* mutant (hereafter referred to as *mtxnpx^−^*). The *mtxnpx^−^* mutant was further manipulated in order to obtain a parasite line with restored mTXNPx expression. This was achieved by integrating the *mTXNPx* ORF into the small sub-unit rRNA *locus* using the pSSU-*PHLEO*-*infantum* vector (Figure 1A), which is a modified version of pSSU-*NEO*-*infantum* [21]. The rescued knockout mutants, designated *mtxnpx^−^*/+*mTXNPx* (Δ*mtxnpx::NEO*/Δ*mtxnpx::HYG* [pSSU-*PHLEO*-*infantum*-*mTXNPx*]), were confirmed by PCR to have the *mTXNPx* ORF and the *PHLEO* cassette correctly integrated into the ribosomal *locus* (Figure 1C). Western blot and indirect immunofluorescence analysis showed that *mtxnpx^−^* mutants lack mTXNPx expression and that the rescued *mtxnpx^−^*/+*mTXNPx* parasites express mTXNPx in its correct subcellular compartment, the mitochondrion (Figures 1D and E).

### mTXNPx is redundant in the insect stage of *L. infantum*

The *mtxnpx^−^* parasite line was generated in the promastigote stage and when grown under standard culture conditions, *i.e.* in serum supplemented RPMI medium at 25°C, it appeared morphologically normal (data not shown) and displayed growth rates similar to that of wild type promastigotes. Most notably, no defects in kinetoplast morphology and division were noticed (Figure 1E). These observations argue against the involvement of mTXNPx in kDNA replication, a function previously attributed to mitochondrial TXNPxs [20], [22]. Even though these results indicate that mTXNPx is redundant in the insect form of *L. infantum*, they do not discard a relevant role for this enzyme during the amastigote stage.

### mTXNPx is crucial for the long term survival of *L. infantum* in the mammalian host

**Figure 2:**
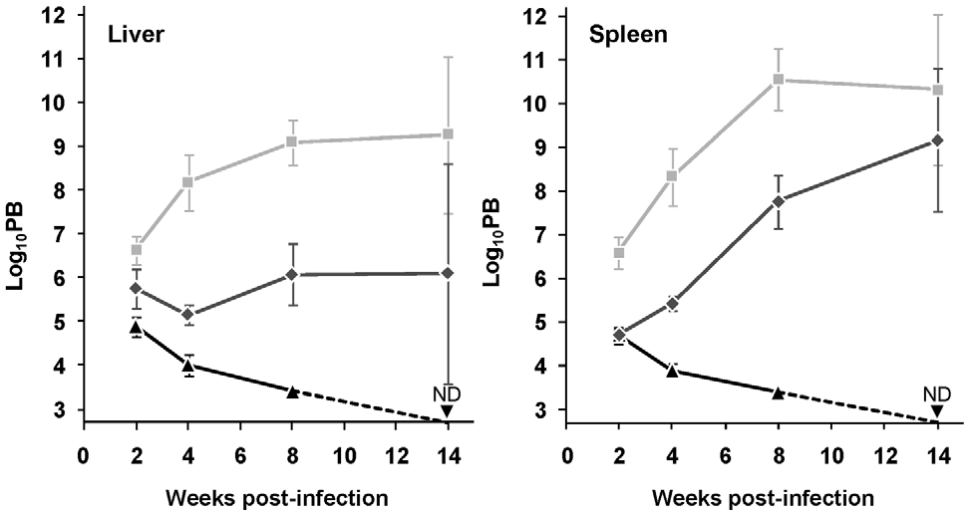
Depletion of mTXNPx impairs *Leishmania* virulence. Wild type (light grey squares), *mtxnpx^−^* (black triangles) and *mtxnpx^−^/+mTXNPx* (dark grey diamonds) promastigotes were inoculated intraperitoneally into BALB/c mice. At different time points after infection, parasite burden (PB) was determined by the limiting dilution assay, as the number of parasites per gram of liver (*left*) or spleen (*right*). The Y axis indicates log_10_PB. Data represent mean and standard error of the mean of 6 independent experiments (involving a total of 85 animals infected with *mtxnpx^−^*), except for *mtxnpx^−^/+mTXNPx*-infected mice, which refer only to 4 experiments. ND, not detected. From week 4 onward, differences between *mtxnpx^−^* and controls are statistically significant. Statistical analysis of these results is in TextS1 and Table S2.

The consequence of mTXNPx depletion on the survival of *L. infantum* amastigotes was assessed by inoculating BALB/c mice, an animal model for visceral leishmaniasis (VL), with equal numbers of stationary phase *mtxnpx^−^*, wild type or *mtxnpx^−^*/+*mTXNPx* promastigotes. At defined time points after infection, parasite loads in the liver and spleen (the organs preferentially infected by VL strains) were analyzed by the limiting dilution assay (LDA). Results in Figure 2 show that elimination of mTXNPx had a remarkable effect on the outcome of infection and that this aggravated over time. Indeed, whilst 2 weeks after infection *mtxnpx^−^* could still be recovered from both infected organs, at 4 and 8 weeks the *mtxnpx^−^* infection indexes were much lower than those of wild type parasites, being below the detection limit of the assay (2.7 log units) at 14 weeks. The impaired virulence of *mtxnpx^−^* was recovered in knockout parasites with restored mTXNPx expression (*mtxnpx^−^*/+*mTXNPx*), confirming that it specifically results from depletion of this enzyme (Figure 2). Of notice, the parasitemia levels of mice infected with *mtxnpx^−^*/+*mTXNPx* were consistently below those produced by wild type parasites, a phenomenon that is frequently observed in complemented knockout mutants [23]–[25]. This is likely due to the fact that in the rescued mutants *mTXNPx* expression is not under the control of its own untranslated regions (as occurs in wild type parasites), but rather it is regulated by the rRNA promoter of the small sub-unit rRNA and by the intergenic region of the cysteine proteinase B 2.8 gene cluster [26].

In short, these results show that mTXNPx is crucial for the long term survival of *L. infantum* amastigotes in the mammalian host. The impaired capacity of mTXNPx-depleted parasites to thrive in mice is not due to their failure to invade host cells (the macrophages) or to differentiate into amastigotes, as inferred from microscopic observation of monolayers of peritoneal macrophages from C57/BL6 mice infected with *mtxnpx^−^* parasites (Figure S1). The next question is which function, disturbed in mutant parasites, is leading to such defect in infectivity.

### Depletion of mTXNPx has no impact on the parasite antioxidant capacity

**Figure 3:**
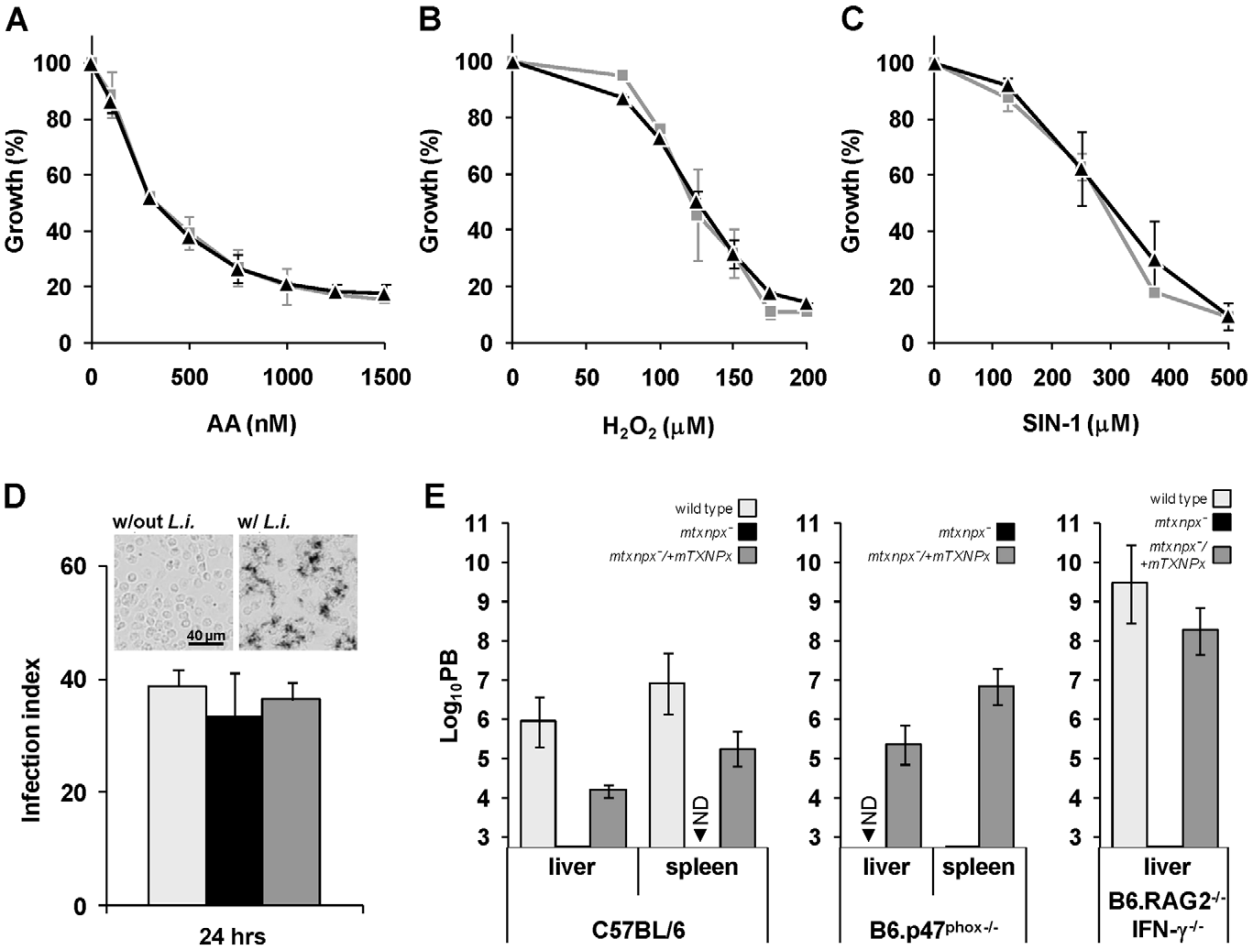
Depletion of mTXNPx has no impact on the antioxidant capacity of promastigotes. A. B. C. Wild type (light grey squares) and *mtxnpx^−^* (black triangles) promastigotes were cultured in the presence of increasing concentrations of (A) antimycin A (AA), (B) H_2_O_2_ or (C) the peroxynitrite donor 3-morpholinosydnonimine hydrochloride (SIN-1). Four days later cell densities were measured in a spectrophotometer at 600 nm. The data is expressed as the percentage of promastigote replication relative to control cultures without any exogenous agent. Graphs represent means and standard deviation of three or more experiments (each performed in duplicate). D. Monolayers of peritoneal macrophages from C57BL/6 mice were infected with stationary phase wild type (light grey bars), *mtxnpx^−^* (black bars) or *mtxnpx^−^/+mTXNPx* (dark grey bars) promastigotes and 24 hrs later the infection indexes determined. The *inset* shows light microscopy photographs of macrophages incubated with NBT in the absence (“w/out *L.i.*”) or presence (“w/*L.i.*”) of parasites. Deposits of dark blue insoluble formazan resulting from NBT reaction with superoxide anion were visible in macrophages 30 min after contact with *L. infantum* promastigotes (*right panel*), but not in macrophages to which no parasites were added (*left panel*). Images were acquired at 100× magnification. E. Mice from different strains (C57BL/6, B6.p47^phox−/−^ and B6.RAG2^−/−^ IFN-γ^−/−^) were inoculated intraperitoneally with wild type (light grey bars), *mtxnpx^−^* (black bars) or *mtxnpx^−^/+mTXNPx* (dark grey bars) stationary phase promastigotes. Five weeks after infection parasite burden (PB) was determined in the livers and spleens of mice by the limiting dilution assay. The Y axis represents log_10_PB. Data represent mean and standard error of the mean of 3 independent experiments involving a total of 72 animals. ND, not detected. Differences between *mtxnpx^−^* and controls are statistically significant. Statistical analysis of these results is in TextS1 and Table S3.

The *L. infantum* mTXNPx is an efficient reductase of hydroperoxides [27] as well as of peroxynitrite (the apparent second order rate for peroxynitrite reduction by mTXNPx is 1.6×10^6^ M^−1^ s^−1^, at pH 7.4 and 37°C; Romao S, Radi R and Tomás AM, unpublished results). As suggested previously [7], [8], [11], the most likely function for mTXNPxs is the elimination of peroxides generated as a consequence of the parasite's aerobic metabolism. Accordingly, *mtxnpx^−^* mutants were assessed for their sensitivity to antimycin A (AA), an inhibitor of the mitochondrial electron transport chain that leads to the local production of reactive oxygen species [28]. However, the observation that AA had the same growth inhibitory effect in *mtxnpx^−^* and wild type promastigotes (Figure 3A) suggests that mTXNPx is not critical for the elimination of peroxides generated within the mitochondrion. A different explanation for the impaired infectivity of *mtxnpx^−^* could be their inability to deal with host-derived oxidants [11]. However, this premise found no support in the observation that susceptibility of *mtxnpx^−^* promastigotes to H_2_O_2_ (added as bolus) or to the peroxynitrite donor 3-morpholinosydnonimine hydrochloride (SIN-1) was similar to that of wild type parasites (Figures 3B and C). This hypothesis was explored further by exposing *mtxnpx^−^* to host-derived oxidants. As shown in the NBT assay in Figure 3D (*inset*), phagocytosis of *L. infantum* promastigotes by murine peritoneal macrophages triggers the generation of superoxide anion (O_2_^.−^), a precursor of H_2_O_2_. The absence of mTXNPx did not render promastigotes more sensitive to such oxidative burst, as deduced from the comparison of the infection indexes of macrophages inoculated with *mtxnpx^−^* and control parasites (wild type and *mtxnpx^−^*/+*mTXNPx*) (Figure 3D). Additional evidence that mTXNPx is not implicated in *Leishmania* protection against host-derived oxidants was obtained in an *in vivo* infection experiment using mutant mice with impaired pro-oxidant capacity, namely B6.p47^phox−/−^ and B6.RAG2^−/−^ IFN-γ^−/−^ mice. B6.p47^phox−/−^ mice carry a targeted disruption of the p47 subunit of the phagocyte NADPH oxidase (phox) complex that is responsible for phagocytosis-induced production of O_2_^.−^. B6.RAG2^−/−^ IFN-γ^−/−^ mice lack B and T cells and do not express interferon gamma (IFN-γ), *i.e.* a pro-inflammatory cytokine predominantly secreted by type-1 T cells that activates the inducible nitric oxide synthase (iNOS) and up-regulates phox, thus allowing for peroxynitrite generation in macrophages. Mutants and control mice of the same genetic background (C57BL/6) were infected with equal numbers of *mtxnpx^−^* and of control parasites (wild type and *mtxnpx^−^*/+*mTXNPx*) and 5 weeks later the parasite burden was analyzed. The results, plotted in Figure 3E, show that the number of *mtxnpx^−^* parasites recovered from the organs of infected mice was either consistently below that of control parasites or undetectable irrespective of the mouse strain serving as host. In other words, even when inoculated in hosts that are unable to mount an efficient oxidative response, *mtxnpx^−^* do not recover infectivity. This observation reinforces the idea that the essential function of mTXNPx in amastigotes is not to provide protection against peroxides of exogenous origin. Additionally, it indicates that the avirulent phenotype of *mtxnpx^−^* is not due to any microbicidal component induced by the host adaptive immune system, namely by the T lymphocytes. Rather, the inability of *mtxnpx^−^* to thrive in mammalian hosts appears to be a consequence of factors inherent to the parasite.

### The critical function of mTXNPx is independent of its peroxidase activity

**Figure 4:**
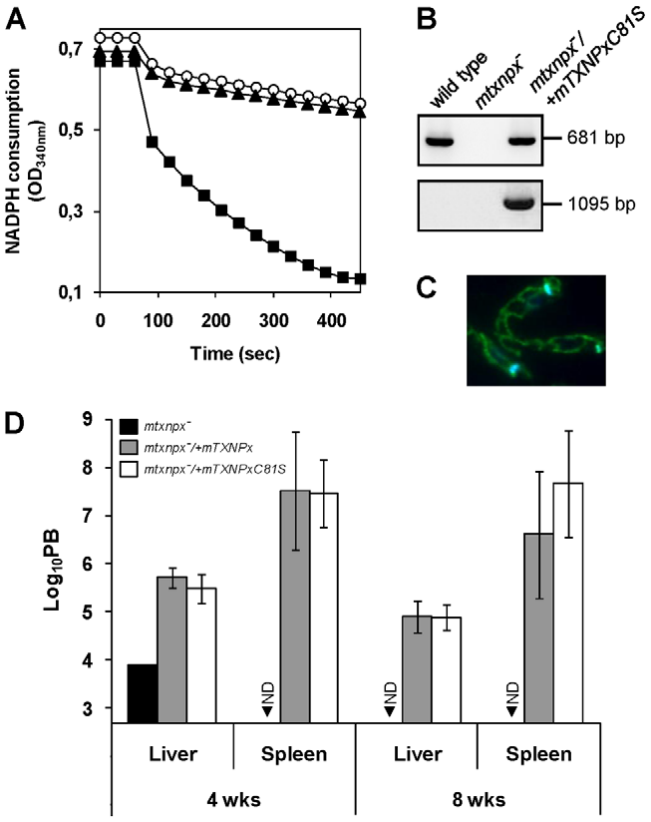
The peroxidase activity of mTXNPx is not a critical determinant of *L. infantum* virulence. A. Classical assay for TXNPx enzymatic activity. Reaction mixtures contained 200 µM NADPH, 0.5 U ml^−1^ *Li*TR, 50 µM TS_2_, 2.5 µM *Li*TXN2 and recombinant TXNPx. ΔmTXNPx was added to a final concentration of 0.5 µM (filled squares) and ΔmTXNPxC81S to 5 µM (filled triangles). Reactions were started by addition of 70 µM H_2_O_2_ at 60 sec and peroxidase activity followed by monitoring NADPH consumption at 340 nm. The negative control contains no TXNPx (open circles). B. PCR analysis of genomic DNA from *mtxnpx^−^/+mTXNPxC81S* parasites using specific primers to amplify the *mTXNPx* ORF (681 bp) or to diagnose for the correct integration of the *PHLEO* cassette into the ribosomal *locus* (1095 bp; location of primers in Figure 1A). Controls, performed with genomic DNA from wild type and *mtxnpx^−^* parasites, are also included. C. Indirect immunofluorescence of *mtxnpx^−^/+mTXNPxC81S* parasites incubated with the anti-mTXNPx antibody (green labeling), merged with DAPI (blue labeling). Parasites were photographed at 1000× magnification. D. Parasite burden (PB) in liver and spleen of BALB/c mice, determined by the limiting dilution assay, 4 and 8 weeks after infection with *mtxnpx^−^* (black bars), *mtxnpx^−^/+mTXNPx* (dark grey bars) or *mtxnpx^−^/+mTXNPxC81S* (open bars) stationary phase promastigotes. The Y axis represents log_10_PB. Data indicate mean and standard error of the mean of 2 independent experiments involving a total of 54 animals. ND, not detected. Differences between *mtxnpx^−^* and controls are statistically significant. Statistical analysis of these results is in TextS1 and Table S4.

Results in the previous section suggested that the crucial function played by mTXNPx during infection is not that of an antioxidant device. To definitively conclude about the contribution of mTXNPx peroxidase activity to *Leishmania* infectivity, a peroxidase-inactive variant of the enzyme (mTXNPxC81S) was tested for its ability to rescue the avirulent phenotype of the knockouts. Cys81 is the peroxidatic Cys of mTXNPx and its replacement by a serine should abolish peroxidase activity, as observed previously [29], [30]. Loss of peroxidase activity of the C81S variant was confirmed *in vitro* using a recombinant enzyme lacking the first 26 amino acids that compose the mitochondrial targeting peptide (ΔmTXNPxC81S) to mimic the mature protein in the parasite's mitochondrion [8]. As expected from previous mutational analysis [29], [30], when assayed for TXNPx activity, the ΔmTXNPxC81S mutein was unable to catalyze H_2_O_2_ reduction, even when present at a 10-fold higher concentration than the control wild type ΔmTXNPx enzyme (Figure 4A). Of notice, on the basis of circular dichroism spectra, substitution of the peroxidatic Cys to a Ser did not alter the secondary structure of the mutein relative to the wild type enzyme, the estimation of α-helix and β-strand contents for both enzymes being of 35% and 29%, respectively (data not shown).

To test whether expression of mTXNPxC81S could restore infectivity of mTXNPx knockouts, *mtxnpx^−^*/+*mTXNPxC81S* mutants were generated by transfecting *mtxnpx^−^* with pSSU-*PHLEO*-*infantum-mTXNPxC81S*. Confirmation for the correct integration of the construct was obtained by PCR (Figure 4B) and the exact subcellular location verified by indirect immunofluorescence analysis (Figure 4C). The *mtxnpx^−^*/+*mTXNPxC81S* transfectants were then inoculated into BALB/c mice and the parasite burden in livers and spleens evaluated at 4 and 8 weeks post infection. The results, depicted in Figure 4D, show that virulence was recovered in *mtxnpx^−^* complemented with mTXNPxC81S and that the parasite load produced by this line was similar to that of *mtxnpx^−^*/+*mTXNPx*. These observations thus provide solid evidence that the essential role played by mTXNPx during the infective stage of *Leishmania* is independent of its peroxidase activity.

### Is mTXNPx functioning *in vivo* as a molecular chaperone?

In addition to acting as peroxidases, 2-Cys PRXs may in some cases exhibit chaperone activity [5], [6], an attribute that, to date, has never been described for any TXNPx. The possibility of mTXNPx acting as a chaperone was therefore investigated as an attempt to provide an alternative function for this enzyme in *Leishmania* amastigotes.

**Figure 5:**
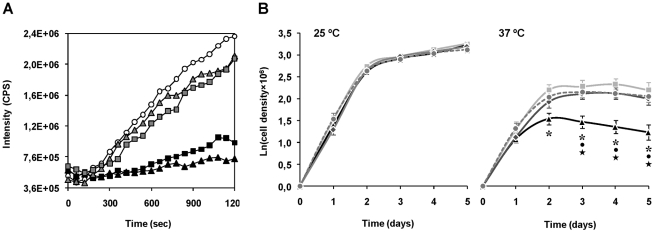
Evidences for the chaperone activity of mTXNPx. A. Chaperone activity of purified recombinant ΔmTXNPx and ΔmTXNPxC81S was analyzed *in vitro* by assessing the enzymes' ability to suppress the thermal aggregation of citrate synthase (CS). Aggregation of CS was induced at 43°C and monitored by measuring light scattering at 500 nm in the absence (open circles) or presence of 5∶1 (grey-filled squares) or 10∶1 (black squares) ratios of ΔmTXNPx monomers to CS monomers. Alternatively, the mutein ΔTXNPxC81S was added to the reaction at 5∶1 (grey-filled triangles) or 10∶1 (black triangles) ratios. B. Wild type (light grey squares), *mtxnpx^−^* (black triangles) *mtxnpx^−^/+mTXNPx* (dark grey diamonds) and *mtxnpx^−^/+mTXNPxC81S* (dark grey circles and dashed line) *L. infantum* promastigotes were seeded at 10^6^ ml^−1^ in complete RPMI medium and incubated at 25°C (*left*) and 37°C (*right*). The Y axis represents ln (or log*_e_*) of cell densities recorded throughout 5 days. Values represent mean and standard error of the mean of 4 to 8 independent curves. Also indicated are the statistically significant differences (i.e. *p*<0.05) between *mtxnpx^−^* and each of the control parasite lines, *i.e.* wild type (*), *mtxnpx^−^/+mTXNPx* (•) and *mtxnpx^−^/+mTXNPxC81S* (★).

The chaperone activity of purified recombinant ΔmTXNPx was assessed *in vitro* by testing its ability to suppress the aggregation of thermally denatured citrate synthase (CS). When incubated at 43°C citrate synthase unfolds, leading to significant aggregation that can be monitored by measuring light scattering in a spectrofluorometer [31]. Addition of ΔmTXNPx to the reaction at a 10-fold molar excess completely suppressed the thermal aggregation of CS (Figure 5A). This effect was much less pronounced when ΔmTXNPx was added at 5-fold molar excess. Importantly, the ΔmTXNPxC81S mutein was also capable of preventing CS aggregation, exhibiting the same behavior as the wild type enzyme (Figure 5A).

To gain insight into the physiological significance of the chaperone activity of mTXNPx a thermotolerance assay was conducted. The experiment consisted in monitoring cell growth of *mtxnpx^−^* and control parasites (wild type, *mtxnpx^−^*/+*mTXNPx*, and *mtxnpx^−^*/+*mTXNPxC81S*) at both 25°C and 37°C, which are the temperatures encountered by the parasite in the insect vector and in the mammalian host, respectively. The cell growth curves depicted in Figure 5B show that, while at 25°C the proliferation rates of all parasite lines were indistinguishable, at 37°C the growth rate of *mtxnpx^−^* was significantly lower than that of wild type parasites. The thermo-sensitive phenotype observed for *mtxnpx^−^* was recovered in the rescued *mtxnpx^−^*/+*mTXNPx* and *mtxnpx^−^*/+*mTXNPxC81S* mutants. These observations show that expression of mTXNPx renders promastigotes more permissive to 37°C, *i.e.* this enzyme confers thermotolerance to *L. infantum* irrespective of its peroxidase activity. It is thus reasonable to speculate that the crucial role played by mTXNPx during amastigote development in the mammalian host is that of a molecular chaperone.

## Discussion

In parasites of the family Trypanosomatidae the peroxidase activity of 2-Cys PRXs (which in these organisms are designated TXNPxs) is accepted as the basis of their physiologic functions [1], [3]. Data presented in this report demonstrate that this activity is not crucial in the case of mTXNPx, even though the protein itself is essential for *L. infantum* amastigote survival. Instead, experimental evidence is provided suggesting that it may be the activity of mTXNPx as a molecular chaperone, unraveled here, that is determinant for parasite viability.

The finding that a peroxidase-inactive version of mTXNPx is competent to ensure amastigote survival excludes *per se* a crucial role for the peroxidase-related functions previously attributed to mTXNPxs, namely elimination of peroxides and regulation of H_2_O_2_-induced apoptosis [7], [8], [11], [18]. Accordingly, these activities must be taken over by other means. Detoxification of exogenously-derived peroxides (such as those generated by the host immune response) might be efficiently fulfilled by cytosolic 2-Cys PRXs and non-selenium glutathione peroxidase-like enzymes (nsGPX) [7], [8], [11], [32]–[34]. As for reduction of peroxides of mitochondrial origin, different scenarios can be envisaged. One possibility is that mitochondrial nsGPX carries out this antioxidant function. This hypothesis requires that the substrate specificities of nsGPX and mTXNPx overlap [33]–[35], and implies the existence of an efficient nsGPX reductant in the mitochondrion, which is still to be found [36]. Of notice, in the particular case of H_2_O_2_, this could diffuse and be reduced by cytosolic peroxidases. In a different perspective, elimination of peroxides may occur via non-catalytic pathways. In this situation, low molecular weight thiols, such as trypanothione (the trypanosomatids' specific thiol), glutathione, ovothiol, free cysteines and even thiol groups exposed on protein surfaces would directly reduce peroxides [37]–[39]. In either circumstance, an ascorbate peroxidase, located in the intermembrane space of the *Leishmania* mitochondrion [40] (gene LinJ.34.0070 in the *L. infantum* GeneDB), may also contribute to peroxide elimination in this organelle.

A different role previously attributed to mTXNPxs is the regulation of kDNA replication. As proposed by Sela *et al.* [20], mTXNPx would affect kDNA replication by oxidizing UMSBP and, consequently, turning “off” the binding of this protein to the minicircle origin of replication. However, in this report no evidence could be found linking mTXNPx depletion to defects in replication rate or in kDNA morphology of *L. infantum* promastigotes. Even though these analyses could not be extended to amastigotes, kDNA replication is highly conserved in trypanosomatids [19], making it difficult to accept that mTXNPx would regulate kDNA replication only in this life stage. Moreover, the proposed model for kDNA replication established a fundamental role for a mitochondrial TXN as reductant of UMSBP and it was shown previously that such enzyme is not essential in *Leishmania* and does not exist in trypanosomes [36]. The mechanism underlying regulation of kDNA replication involving mTXNPxs (and a mitochondrial TXN) must, therefore, be re-evaluated.

This work has also, for the first time, uncovered that trypanosomatid 2-Cys PRXs can function as molecular chaperones and that this activity might be relevant for parasite survival *in vivo*. Several pieces of evidence point into this direction. i) Purified recombinant mTXNPx was shown to prevent the thermal aggregation of CS, a well-known chaperone substrate [31]. This activity does not depend on the peroxidatic capacity of mTXNPx, as the mTXNPxC81S mutein was also active in this assay, thus providing a rationale for the recovery of virulence observed for *mtxnpx^−^*/+*mTXNPxC81S*. This is in line with observations reported by Jang *et al.* [5]. ii) The chaperone activity of 2-Cys PRXs is known to depend on their quaternary structure and, as shown before, mTXNPx is capable of associating into decameric ring-like structures [27], which are the minimal oligomeric arrangements required for such function [41]. iii) Gain of chaperone function and loss of peroxidase activity of 2-Cys PRXs are, in most organisms, accompanied with the overoxidized form of the protein [2] and mTXNPx undergoes overoxidation when promastigotes are exposed to 37°C (Figure S2). Since *Leishmania* do not possess sulfiredoxins to enzymatically regenerate overoxidized 2-Cys PRXs [1] as occurs in higher eukaryotes, such peroxidase-inactive form of mTXNPx is likely to persist until the protein is synthesized *de novo*. iv) Additional support consubstantiating a role for mTXNPx as a chaperone came from the observation that lack of mTXNPx expression rendered promastigotes more sensitive to 37°C, the temperature encountered in the mammalian host. Such thermo-sensitive phenotype is reminiscent of some chaperone-deficient mutants [42]–[44]. Accepting that mTXNPx is a molecular chaperone *in vivo*, one can only speculate how this function could impact on parasite survival under the conditions met in the host. In mitochondria, molecular chaperones are associated with a multitude of functions. They participate in the biogenesis of new mitochondrial peptides by assisting their trans-membrane transport and their refolding to the native conformation inside the matrix. In addition, they are part of the mitochondrial protein quality control system that repairs damaged or misfolded proteins or mediates their removal by proteolysis [45]. Under situations of cellular stress (including elevated temperatures), the activity of mitochondrial chaperones becomes even more imperative for protein homeostasis [45]. Accordingly, it is reasonable to assume that mTXNPx functions as a molecular chaperone that ensures integrity of mitochondrial functions, its activity being particularly relevant when parasites reside in vertebrate host.

Another important outcome of this report is the identification of mTXNPx as a new factor critical for *Leishmania* infectivity. This finding may have impact on the development of new strategies to control leishmaniasis as the *mtxnpx^−^* parasite line produced here can be regarded as the basis of a live attenuated vaccine. In this context, the observation that *mtxnpx^−^* cannot give rise to a productive infection even in immunocompromised mice, suggests that this strategy would not pose major safety issues.

In conclusion, the work presented in this manuscript sheds lights on the functional relevance of mTXNPx by showing that, even though this molecule is crucial for *L. infantum* survival during infection of the vertebrate host, its peroxidase activity is superfluous. A novel peroxidase-unrelated function, as a molecular chaperone, is proposed for this enzyme, which could be critical for mitochondrial functionality under the conditions encountered by the parasite in the mammalian host. This report thus constitutes a turning point into the current state of knowledge regarding the physiologic role of peroxiredoxins in trypanosomatids.

## Materials and Methods

### Ethics statement

The experimental animal procedures were approved by the Local Animal Ethics Committee of Institute for Molecular and Cell Biology, University of Porto, Portugal and licensed by DGV (General Directory of Veterinary, Ministry of Agriculture, Rural Development and Fishing, Govt. of Portugal), in May 18, 2006 with reference 520/000/000/2006. All animals were handled in strict accordance with good animal practice as defined by national authorities (DGV, Law nu1005/92 from 23rd October) and European legislation EEC/86/609.

### Parasite cultures

*Leishmania infantum* promastigostes (MHOM MA67ITMAP263) were cultured at 25°C in RPMI 1640 Glutamax medium, supplemented with 10% inactivated fetal bovine serum (FBSi), 50 U ml^−1^ penicillin, 50 µg ml^−1^ streptomycin (all from Gibco) and 25 mM hepes sodium salt pH 7.4 (Sigma).

### Generation of knockout and rescue constructs

Primers used to generate the constructs are summarized in Table S1. The accuracy of all assembled constructs was verified by sequencing. To produce the *NEO* replacement construct, sections of the 5′ and 3′ non-coding sequences flanking the *mTXNPx* ORF were PCR-amplified from a cosmid clone [8] using primers P1/P2 and P3/P4, respectively. Following digestion with the appropriate restriction enzymes, PCR products were cloned into the *Bam*HI-*Xho*I and *Kpn*I sites of the pTEX-*NEO* plasmid [46], on both sides of the neomycin phosphotransferase (*NEO*) gene. To assemble the *HYG* disruption construct, fragments containing part of the 5′ and 3′ untranslated regions and of the *mTXNPx* coding sequence were amplified by PCR from genomic DNA of the *NEO*-targeted mutants, using primers P5/P6 and P7/P8. Upon digestion with the appropriate restriction enzymes, PCR products were cloned into *Bam*HI-*Eco*RV and *Kpn*I sites of the pTEX-*HYG* plasmid, a version of pTEX-*NEO* wherein the *NEO* gene had been replaced by the hygromycin phosphotransferase (*HYG*) open reading frame. To generate the rescue construct, the *mTXNPx* ORF was introduced into the *Xma*I and the Klenow-treated *Bam*HI restriction sites of the pSSU-*NEO*-*infantum* plasmid [21]. This plasmid is a modified version of the pSSU-int [26], wherein the *HYG* gene had been replaced by *NEO* and the 5′ flanking sequence of the small sub-unit rRNA (18S rRNA) gene of *Leishmania mexicana* substituted by the homologous sequence of *L. infantum*. Upon cloning of the *mTXNPx* coding sequence into pSSU-*NEO*-*infantum*, the *NEO* ORF was replaced by the bleomycin hydrolase (*PHLEO*) gene. One additional rescue construct (pSSU-*NEO*-*infantum-mTXNPxC81S*), containing a mutated version of *mTXNPx*, was generated by site directed mutagenesis (see below). Before transfection of *L. infantum* promastigotes, the *NEO*, *HYG* and *PHLEO* constructs were linearized by digestion with *Hinc*II, *Bam*HI-*Sac*I, and *Nde*I-*Pme*I, respectively, and purified from agarose gels by electroelution.

### Production of the mTXNPxC81S mutein

A site directed mutagenesis strategy was employed to introduce the Cys81 to Ser (C81S) mutation in the *mTXNPx* ORF cloned into pSSU-*PHLEO*-*infantum* (detailed above) and pET28c (detailed below). For this, the full-length plasmids were PCR-amplified with sense and antisense mutated primers (P9 and P10 in Table S1) using *Pfu* polymerase (Stratagene). Upon digestion of parental (wild type) DNA with 10 units of *Dpn*I (New England Biolabs) for 1 h at 37°C, the reaction (4 µl) was used directly to transform *Escherichia coli* DH5α strain. Mutant colonies were confirmed to carry the site-directed mutation by sequencing both strands.

### Transfection of *L. infantum* and isolation of mutants

Promastigotes in the logatithmic phase of growth were electroporated at 450 V and 350–400 µF with 1 to 5 µg of DNA as described elsewhere [47]. Parasites were allowed to recover in 10 ml of culture medium without selective drugs for 24 hours. Drugs were then added to 5 ml of the liquid culture and the remaining 5 ml were pelleted and plated onto agar plates containing the same drug(s). Geneticin (G418; Sigma) was used at 15 µg ml^−1^, hygromycin (Invitrogen) at 10 µg ml^−1^ and bleomycin (Sigma) at 17.5 µg ml^−1^. Upon 2 to 3 weeks of growth on agar, colonies were picked up and transferred into liquid medium.

### Indirect immunofluorescence assay (IFAT)

Immunofluorescence assays were performed according to Castro *et al.* [8]. Briefly, parasites were fixed with 4% paraformaldehyde (w/v) in PBS (0.1 M sodium phosphate buffer pH 7.2, 0.15 M NaCl), spotted onto polylysine-coated microscope slides, permeabilized with 0.1% (v/v) Triton X-100 and incubated with the polyclonal anti-mTXNPx antibody [8] and 4′,6-diamidino-2-phenylindole (DAPI). The secondary antibody was Alexa Fluor 488 anti-rabbit IgG (Molecular Probes). Slides were mounted in Vectashield (Vector Laboratories) and examined with an AxioImager Z1 microscope (Carl Zeiss, Germany).

### Drug sensitivity assays

*L. infantum* promastigotes in the logarithmic phase of growth were seeded at 10^6^ cells ml^−1^ in 24-well plates containing increasing concentrations (in duplicate) of either antimycin A (AA), H_2_O_2_ or 3-morpholinosydnonimine hydrochloride (SIN-1) (all from Sigma). Parasites were allowed to grow for 3–4 days and cell densities were measured in a spectrophotometer at 600 nm.

### Animals and ethics statement

BALB/c, C57BL/6 and National Marine Research Institute (NMRI) mice were purchased from Charles River (Madrid, Spain). Mice with a targeted disruption of the p47 subunit of the NADPH-oxidase complex on a C57BL/6 background (B6.p47^phox−/−^) were purchased from Taconic (Lille Skensved, Denmark). Until the day of infection with *L. infantum*, and as prophylatic treatment against bacterial infection, trimethoprim-sulfamethoxazole (Bactrim; 600 mg l^−1^) was administered in the drinking water to B6.p47^phox−/−^ mice. Mice double deficient in gamma interferon (IFN-γ) and in recombinase activating gene-2 (RAG-2) were obtained by crossing single knock out strains on C57BL/6 background. All mice were raised in specific pathogen-free conditions. Euthanasia was performed in a 20% isofluorane atmosphere and all efforts were made to minimize suffering.

### Infection of monolayers of murine macrophages with promastigotes

Macrophages obtained by peritoneal lavage of C57/BL6 mice were seeded at 4×10^5^ cells per well in DMEM Glutamax complemented with 10% FBSi, 50 U ml^−1^ penicillin and 50 µg ml^−1^ streptomycin (all from Gibco), and allowed to adhere for 2 hrs at 37°C, 5% CO_2_. Upon washing with Hanks balanced salt solution (HBSS, Gibco), macrophages were incubated with opsonized promastigotes (in the stationary phase of growth) at a parasite:macrophage ratio of 10∶1. For detection of superoxide anion (O_2_·^−^), a freshly prepared solution of 1 mg ml^−1^ nitroblue tetrazolium (NBT), prepared in Dulbecco's PBS was added to macrophages. After 30 min of incubation at 37°C, cells were washed with HBSS, fixed with methanol and deposition of formazan observed with an optical microscope Olympus CX31. For determination of infection indexes, infection with promastigotes was allowed to proceed for 3 hrs, after which non-internalized parasites were removed with HBSS. Cells were cultured for additional 24 hrs and then washed, fixed with 4% paraformaldehyde (w/v) in PBS, permeabilized with 0.1% (v/v) Triton X-100, and incubated with the anti-cTXNPx1 [8] antibody and propidium idodide (PI). Secondary antibody was Alexa Fluor 488 anti-rabbit IgG (Molecular Probes). Slides were mounted in Vectashield (Vector Laboratories). Images were acquired with an Axiocam MR ver.3.0 camera (Carl Zeiss, Germany) coupled to an AxioImager Z1 microscope (Carl Zeiss, Germany). A minimum of 3000 macrophages was counted in each experiment using specifically designed software (unpublished data) and the infection index was obtained by multiplying the percentage of infected macrophages by the average number of intracellular amastigotes per macrophage.

### Determination of parasite burden by the limiting dilution assay

Parasites of all lines were passaged through NMRI mice at least twice prior to infection experiments. Next, 10^8^ *L. infantum* stationary phase promastigotes, were inoculated intraperitoneally into 6- to 9-weeks old male BALB/c mice. At defined time points, mice were sacrificed and their livers and spleens excised, weighed and homogenized in Schneider's medium (Sigma) supplemented with 10% FBSi, 100 U ml^−1^ penicillin, 100 µg ml^−1^ streptomycin (all from Gibco), 5 mM hepes sodium salt pH 7.4 (Sigma), 5 µg ml^−1^ phenol-red (Sigma) and 2% sterile human urine. Homogenates were then diluted to 10 mg ml^−1^ and these cell suspensions titrated in quadruplicate across a 96-well plate in serial four-fold dilutions (four titrations per organ). After two weeks of growth at 25°C, the last dilution containing promastigotes was recorded and the number of parasites per gram of organ (parasite burden) calculated as described by Buffet *et al.* [48]. The detection limit of this method is 500 parasites g^−1^ (*i.e.* 2.7 log units).

### Heterologous expression and purification of ΔmTXNPx and ΔmTXNPxC81S

To mimic the mature mitochondrial protein, truncated wild type mTXNPx (ΔmTXNPx), *i.e.* lacking the first 26 amino acids that compose the mitochondrial targeting peptide [8], was produced in the *E. coli* strain BL21 (DE3) Tuner upon transformation a pET28c (Novagen) construct containing the ΔmTXNPx gene fragment (PCR-amplified with primers P11/P12 in Table S1). To produce the mutant ΔmTXNPxC81S protein, the pET28c-Δ*mTXNPxC81S* was generated by site directed mutagenesis (detailed above) and used to transform the same *E. coli* strain. Both enzymes were expressed in bacteria as fusion proteins carrying an N-terminal six histidine tag. Upon induction for 3 hrs at 30°C in the presence of 50 µg ml^−1^ kanamycin and 0.1 mM isopropyl-*β*-D-thiogalactopyranoside (IPTG), bacteria were pelleted, suspended in 500 mM NaCl, 20 mM Tris-HCl pH 7.6, disrupted by sonication and centrifuged at 30,000×*g* for 30 min at 4°C. The supernatant was applied to a His Bind resin (Novagen) column and the recombinant protein eluted with an imidazole gradient (5 to 1000 mM) at a flow rate of 1 ml min^−1^. Fractions confirmed to contain the protein by SDS-PAGE were pooled, applied to PD-10 columns (Amersham) and eluted in 50 mM sodium phosphate buffer pH 8.0. For removal of the His tag, enzymes were first digested with biotinylated thrombin (Novagen), and then incubated with immobilized streptavidin for removal of the protease. Proteins were concentrated by ultrafiltration, recovered in 40 mM hepes pH 7.5 and quantified by the bicinchoninic acid (BCA) protein assay (Pierce), using bovine serum albumin (BSA) as standard.

### Determination of peroxidase activity

Routine determination of TXNPx activity was performed according to Nogoceke *et al.* [4]. Briefly, reaction mixtures were prepared in a total volume of 300 µl of 50 mM Tris-HCl, 1 mM EDTA, pH 8.0, containing 200 µM NADPH, 0.5 U ml^−1^ *L. infantum* TR, 50 µM trypanothione disulfide (TS_2_, Bachem), 2.5 µM *L. infantum* TXN2 [49] and varying concentrations of *Δ*mTXNPx or *Δ*mTXNPxC81S. Reactions were started by addition of 70 µM hydrogen peroxide (H_2_O_2_, Sigma) and NADPH consumption was followed at 340 nm. All reactions were performed at 25°C and monitored with a Shimadzu UV-2401 PC spectrophotometer (Shimadzu Corporation).

### Determination of chaperone activity

The chaperone activity of ΔmTXNPx and ΔmTXNPxC81S was measured using citrate synthase (CS) from porcine heart (Sigma) as substrate, as described before [31]. Each enzyme was diluted in 40 mM hepes sodium salt pH 7.5 to reach final concentrations of 0.75 or 1.5 µM. A cocktail of protease inhibitors and 100 µM DTT were added to the reaction mixture to prevent degradation of CS and disulfide cross-linking between the peroxiredoxin and CS molecules, respectively, both of which could result in an apparent chaperone-like activity. Samples were pre-incubated at 43°C for 5 min and the reaction started by addition of CS to a final concentration of 0.15 µM. Light scattering due to CS aggregation at 43°C was monitored using a FluoroMax-4 spectrofluorometer, with excitation and emission wavelengths set to 500 nm and excitation and emission slits set to 2 nm. Data were recorded for 20 min.

### Thermotolerance assays

*L. infantum* promastigotes of all lines, previously synchronized by 4–5 daily passages of 5×10^5^ cells ml^−1^, were seeded at 10^6^ ml^−1^ and allowed to grow for 4 days at either 25°C or 37°C. Every 24 hours cell densities were determined with a Neubauer-counting chamber for growth curve determination.

### Statistical analysis

Comparisons between the parasite lines were carried out using analysis of variance. When normality or homogeneity of variances was not observed the Kruskal-Wallis non parametric test was used. In this case, multiple comparisons were carried using the Mann-Whitney test, with the Bonferroni correction. In order to investigate whether the distribution of the negative versus positive organs (in the LDA) was independent of the parasite line, the Chi-square and the Fisher's exact tests were employed (see Text S1). Statistical significance was assessed for p<0.05. The analysis was carried out using IBM SPSS Statistics 19.

## Supporting Information

Figure S1 **mTXNPx depletion has no impact on the capacity of** ***L. infantum*** **to invade macrophages and differentiate into amastigotes.** Fluorescence microscopy images showing monolayers of peritoneal macrophages from C57BL/6 mice 24 hrs after infection with *mtxnpx^−^* (*upper panels*) and with wild type *L. infantum* (*lower panels*). For parasite detection, the anti-cTXNPx1 antibody [8], which specifically recognizes *Leishmania* antigens, was used (*left*). Merging with propidium iodide (PI) is also shown (*right*). Arrows point to intracellular parasites. Images were acquired at a 200× magnification.
(PDF)

Figure S2 **Susceptibility of mTXNPx to overoxidation in promastigotes exposed to 37°C.** *Leishmania infantum* promastigotes grown for 4 days at either 25°C or 37°C (as in Figure 5B) were collected (1.4×10^7^ cells) and lysed in the presence of 200 mM *N*-ethylmaleimide (NEM) (*left*). Alternatively, cell lysates were pre-treated with H_2_O_2_ (50 µM H_2_O_2_ for 30 min, followed by a further addition of 50 µM H_2_O_2_) prior to fixation with NEM (*right*). Extracts were examined by western blot with the anti-mTXNPx antibody. The dimer corresponds to the oxidized form of mTXNPx, whereas the monomer corresponds to either the reduced or the overoxidized protein. In cell lysates of parasites grown at 37°C the monomeric form of mTXNPx persists even upon H_2_O_2_ pre-treatment, indicating that this is the overoxidized (peroxidase inactive) form of the enzyme. This western blot is representative of two independent experiments. Protein loading was controlled by Ponceau staining.
(PDF)

Table S1 **List of oligonucleotides employed to generate DNA constructs.**
(PDF)

Table S2 **Distribution of organs testing negative and positive in the limiting dilution assays used to generate the plot in** **Figure 2** **of the main text.**
(PDF)

Table S3 **Distribution of organs testing negative and positive in limiting dilution assays used to generate the plot in** **Figure 3E** **of the main text.**
(PDF)

Table S4 **Distribution of organs testing negative and positive in limiting dilution assays used to generate the plot in** **Figure 4D** **of the main text.**
(PDF)

Text S1 **Statistical analysis of parasite burden.**
(PDF)
