# Supplementary material for: Leishmania Mitochondrial Peroxiredoxin Plays a Crucial Peroxidase-Unrelated Role during Infection: Insight into Its Novel Chaperone Activity
Source: PLoS Pathog. 2011 Oct 27;7(10):e1002325. doi: 10.1371/journal.ppat.1002325 (PMC3203189; doi:10.1371/journal.ppat.1002325)
Supplement: Figure S1 — mTXNPx depletion has no impact on the capacity of L. infantum to invade macrophages and differentiate into amastigotes. Fluorescence microscopy images showing monolayers of peritoneal macrophages from C57BL/6 mice 24 hrs after infection with mtxnpx− (upper panels) and with wild type L. infantum (lower panels). For parasite detection, the anti-cTXNPx1 antibody [8], which specifically recognizes Leishmania antigens, was used (left). Merging with propidium iodide (PI) is also shown (right). Arrows point to intracellular parasites. Images were acquired at a 200× magnification. (PDF) [file ppat.1002325.s001.pdf]

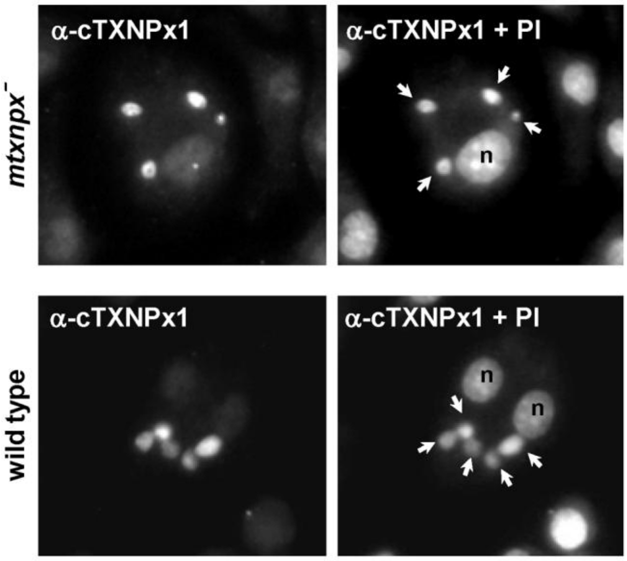

Figure S1. mTXNPx depletion has no impact on the capacity of *L. infantum* to invade macrophages and differentiate into amastigotes.

Fluorescence microscopy images showing monolayers of peritoneal macrophages from C57BL/6 mice 24 hrs after infection with *mtxnpx*$^{-}$ (*upper panels*) and with wild type *L. infantum* (*lower panels*). For parasite detection, the anti-cTXNPx1 antibody [8], which specifically recognizes *Leishmania* antigens, was used (*left*). Merging with propidium iodide (PI) is also shown (*right*). Arrows point to intracellular parasites. Images were acquired at a 200× magnification.
