# Supplementary material for: Leishmania Mitochondrial Peroxiredoxin Plays a Crucial Peroxidase-Unrelated Role during Infection: Insight into Its Novel Chaperone Activity
Source: PLoS Pathog. 2011 Oct 27;7(10):e1002325. doi: 10.1371/journal.ppat.1002325 (PMC3203189; doi:10.1371/journal.ppat.1002325)
Supplement: Figure S2 — Susceptibility of mTXNPx to overoxidation in promastigotes exposed to 37°C. Leishmania infantum promastigotes grown for 4 days at either 25°C or 37°C (as in Figure 5B) were collected (1.4×10^7^ cells) and lysed in the presence of 200 mM N-ethylmaleimide (NEM) (left). Alternatively, cell lysates were pre-treated with H2O2 (50 µM H2O2 for 30 min, followed by a further addition of 50 µM H2O2) prior to fixation with NEM (right). Extracts were examined by western blot with the anti-mTXNPx antibody. The dimer corresponds to the oxidized form of mTXNPx, whereas the monomer corresponds to either the reduced or the overoxidized protein. In cell lysates of parasites grown at 37°C the monomeric form of mTXNPx persists even upon H2O2 pre-treatment, indicating that this is the overoxidized (peroxidase inactive) form of the enzyme. This western blot is representative of two independent experiments. Protein loading was controlled by Ponceau staining. (PDF) [file ppat.1002325.s002.pdf]

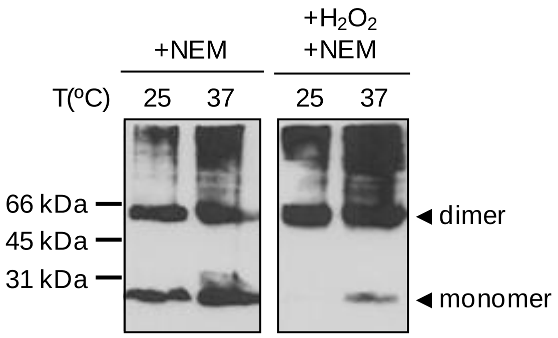

Figure S2. Susceptibility of mTXNPx to overoxidation in promastigotes exposed to 37ºC. *Leishmania infantum* promastigotes grown for 4 days at either 25ºC or 37ºC (as in Figure 5B) were collected (1.4×10^7 cells) and lysed in the presence of 200 mM *N*-ethylmaleimide (NEM) (*left*). Alternatively, cell lysates were pre-treated with H_2O_2 (50 µM H_2O_2 for 30 min, followed by a further addition of 50 µM H_2O_2) prior to fixation with NEM (*right*). Extracts were examined by western blot with the anti-mTXNPx antibody. The dimer corresponds to the oxidized form of mTXNPx, whereas the monomer corresponds to either the reduced or the overoxidized protein. In cell lysates of parasites grown at 37ºC the monomeric form of mTXNPx persists even upon H_2O_2 pre-treatment, indicating that this is the overoxidized (peroxidase inactive) form of the enzyme. This western blot is representative of two independent experiments. Protein loading was controlled by Ponceau staining.
