# Supplementary material for: Leishmania Mitochondrial Peroxiredoxin Plays a Crucial Peroxidase-Unrelated Role during Infection: Insight into Its Novel Chaperone Activity
Source: PLoS Pathog. 2011 Oct 27;7(10):e1002325. doi: 10.1371/journal.ppat.1002325 (PMC3203189; doi:10.1371/journal.ppat.1002325)
Supplement: Table S1 — List of oligonucleotides employed to generate DNA constructs. (PDF) [file ppat.1002325.s003.pdf]

Table S1. List of oligonucleotides employed to generate DNA constructs.

Primer name	Primer sequence
P1	5’-cacc*ggatcc* CTTCGATCAAGTTAACCGCC-3’
P2	5’-caccg*ctcgag* AGACGGCGGAGCATCGTGT-3’
P3	5’-gcgg*ggtacc* ATGTCTTTCACCTATACACATG-3’
P4	5’-acgg*ggtacc* TGTTTGATCTGTCGACTGGG-3’
P5	5’-cgc*gGATCC* GGGTGGCAGTATC-3’
P6	5’-cggag*gatatc* GCTTCTCAAAGTCGGCGT-3’
P7	5’-gcgg*ggtacc* GTGTGCTGATCGAGGAAT-3’
P8	5’-gcgg*ggtacc* GAGCTCAAAAGCTCGCAT-3’
P9	5’-CTTCACCTTCGTTTCCCCGACCGAGATCA-3’
P10	5’-TGATCTCGGTCGGGGAAACGAAGGTGAAG-3’
P11	5’-ccgcgca*catatg* AATCTGGACTATCAGATGTAC-3’
P12	5’-caccg*ctcgag* TCACATGTTCTTCTCGAAAAAC-3’

Clamp sequences are indicated in lower case, restriction sites in italic and mutations used to generate the mTXNPxC81S mutein underlined.
