# Supplementary material for: Leishmania Mitochondrial Peroxiredoxin Plays a Crucial Peroxidase-Unrelated Role during Infection: Insight into Its Novel Chaperone Activity
Source: PLoS Pathog. 2011 Oct 27;7(10):e1002325. doi: 10.1371/journal.ppat.1002325 (PMC3203189; doi:10.1371/journal.ppat.1002325)
Supplement: Table S2 — Distribution of organs testing negative and positive in the limiting dilution assays used to generate the plot in Figure 2 of the main text. (PDF) [file ppat.1002325.s004.pdf]

Table S2. Distribution of organs testing negative and positive in the limiting dilution assays used to generate the plot in Figure 2 of the main text.

Time after infection	Parasite strain	Liver				Spleen			
		LDA result		Statistical test		LDA result		Statistical test	
		Negative	Positive	Chi-square	Fisher´s [a]	Negative	Positive	Chi-square	Fisher´s [a]
2 wks	wild type	17%	83%	n.s.	n.s.	17%	83%	n.s.	n.s.
	mtxnpx^{-}	31%	69%			19%	81%		
	mtxnpx^{-}/+*mTXNPx*	50%	50%			50%	50%		
4 wks	wild type	13%	87%	p<0.05 *	p<0.05	13%	87%	p<0.05 *	p<0.05
	mtxnpx^{-}	71%	29%			50%	50%		
	mtxnpx^{-}/+*mTXNPx*	8%	92%			8%	92%		
8 wks	wild type	15%	85%	p<0.05	p<0.05	15%	85%	p<0.05	p<0.05
	mtxnpx^{-}	95%	5%			95%	5%		
	mtxnpx^{-}/+*mTXNPx*	20%	80%			7%	93%		
14 wks	wild type	0%	100%	p<0.05 *	p<0.05	0%	100%	p<0.05 *	p<0.05
	mtxnpx^{-}	100%	0%			100%	0%		
	mtxnpx^{-}/+*mTXNPx*	40%	60%			20%	80%		

[a] Comparison between *mtxnpx*$^{-}$ and wild type plus *mtxnpx*$^{-}$/+*mTXNPx*; * more than 25 % of cells with expected values <5; n.s., no statistical significance (*i.e.* p>0.05).
