# Supplementary material for: Leishmania Mitochondrial Peroxiredoxin Plays a Crucial Peroxidase-Unrelated Role during Infection: Insight into Its Novel Chaperone Activity
Source: PLoS Pathog. 2011 Oct 27;7(10):e1002325. doi: 10.1371/journal.ppat.1002325 (PMC3203189; doi:10.1371/journal.ppat.1002325)
Supplement: Table S3 — Distribution of organs testing negative and positive in limiting dilution assays used to generate the plot in Figure 3E of the main text. (PDF) [file ppat.1002325.s005.pdf]

Table S3. Distribution of organs testing negative and positive in limiting dilution assays used to generate the plot in Figure 3E of the main text.

Mouse strain	Parasite strain	Liver				Spleen			
		LDA result		Statistical test		LDA result		Statistical test	
		Negative	Positive	Chi-square	Fisher´s [(a)]	Negative	Positive	Chi-square	Fisher´s [(a)]
C57/BL6	wild type	20%	80%	$p<0.05$ *	$p<0.05$	10%	90%	$p<0.05$ *	$p<0.05$
	mtxnpx$^-$	86%	14%			100%	0%		
	mtxnpx$^-$*/+mTXNPx*	10%	90%			10%	90%		
B6.p47$^{phox-/-}$	*mtxnpx*$^-$	100%	0%	$p<0.05$ *	$p<0.05$	75%	25%	$p<0.05$ *	$p<0.05$
	mtxnpx$^-$*/+mTXNPx*	0%	100%			0%	100%		
B6.RAG2$^{-/-}$ IFN-$\gamma^{-/-}$	wild type	11%	89%	$p<0.05$ *	$p<0.05$	n.a.	n.a.	-	-
	mtxnpx$^-$	89%	11%						
	mtxnpx$^-$*/+mTXNPx*	11%	89%						

[(a)] Comparison between *mtxnpx*$^-$ and wild type plus *mtxnpx*$^-$*/+mTXNPx*; * more than 25 % of cells with expected values <5; n.a., not analyzed.
