# Supplementary material for: Leishmania Mitochondrial Peroxiredoxin Plays a Crucial Peroxidase-Unrelated Role during Infection: Insight into Its Novel Chaperone Activity
Source: PLoS Pathog. 2011 Oct 27;7(10):e1002325. doi: 10.1371/journal.ppat.1002325 (PMC3203189; doi:10.1371/journal.ppat.1002325)
Supplement: Text S1 — Statistical analysis of parasite burden. (PDF) [file ppat.1002325.s007.pdf]

Text S1

Statistical analysis of parasite burden

Statistical analysis of parasite burden was carried out taking into account three experimental constraints inherent to the limiting dilution assay (LDA). First, this technique does not allow the detection of parasites in the whole organ (only in a small fraction of the liver and spleen) and, as such, has a detection limit associated to it, which under our experimental settings was 2.7 log units. Accordingly, the LDA does not discriminate between organs that are infected below the detection limit and those that are not infected. Therefore, all organs that were LDA-negative were excluded in the calculation of the average and standard error of the mean used to produce plots in Figures 2, 3E and 4D of the main text. Second, from week 4 p.i. onward, most organs tested negative for the presence of *mtxnpx*$^{-}$ parasites in the LDA. In some of the time points analyzed, only one organ exhibited *mtxnpx*$^{-}$ parasitemia indexes ≥2.7 log units. The limited number of organs testing positive for *mtxnpx*$^{-}$ rendered inadequate the use of the ANOVA or of the t-test for statistical comparison of the different parasite strains. Finally, under our experimental conditions, approximately 13% of the animals infected i.p. with wild type *L. infantum* revealed to be negative in the LDA. This likely resulted i) from the imperfect administration of the parasite inoculum and/or ii) from the fact that some animals resolve infection and clear most, if not all, parasites. In spite of these limitations, the frequency of *mtxnpx*$^{-}$-negative organs was consistently higher than that observed for wild type or for the other parasite strains used in this study. To statistically validate this difference, we employed the Chi-square test (for comparison of more than two parasite strains) or the Fisher's exact test (when only two strains were compared). One limitation found when using the Chi-square test was that, in some cases, no conclusions regarding statistical significance could be made, since the proportion of cells with expected frequencies below 5 was greater than 25%. Whenever this condition was observed wild type and *mtxnpx*$^{-}$-complemented mutants (either *+mTXNPx* or *+mTXNPxC81S*) were grouped and compared with *mtxnpx*$^{-}$ using the Fisher's exact test. The decision to group wild type and *mtxnpx*$^{-}$-complemented strains was based on the observation that the distribution of negative and positive organs in mice infected by these parasite lines was similar (*i.e.* with no statistically significant differences amongst them). The distribution of LDA positive and negative organs for each experiment is

shown in Tables S2-4, as well as the results from the Qui-square and the Fisher's exact tests.

Table S2 shows that from 4 wks p.i. onwards the number of negative organs was higher for *mtxnpx*$^-$-infected mice than for mice infected with control parasites. This difference was statistically validated by the Chi-square and the Fisher's exact tests.

Data on Table S3 indicates that, irrespective of the mouse strain used as host, the number of negative organs was always higher for *mtxnpx*$^-$-infected mice than for mice infected with wild type or with *mtxnpx*$^-$*/+mTXNPx* parasites. This difference was statistically significant according to the Chi-square and the Fisher's exact tests.

As depicted in Table S4, at all times p.i. tested in this experiment, the number of negative organs was always higher for *mtxnpx*$^-$-infected mice than for mice infected with *mtxnpx*$^-$*/+mTXNPx* or with *mtxnpx*$^-$*/+mTXNPxC81S* parasites. This difference was statistically validated by the Chi-square and the Fisher's exact tests.
